# Detection of HIV-1 dual infections in highly exposed treated patients

**DOI:** 10.1186/1743-422X-8-392

**Published:** 2011-08-08

**Authors:** Guadalupe Andreani, Constanza Espada, Ana Ceballos, Juan Ambrosioni, Alejandro Petroni, Dora Pugliese, María Belén Bouzas, Silvia Fernandez Giuliano, Mercedes C Weissenbacher, Marcelo Losso, Jorge Benetucci, Jean K Carr, Liliana Martínez Peralta

**Affiliations:** 1 National Reference Center for AIDS, Microbiology Department, (Paraguay 2155), School of Medicine, University of Buenos Aires, Buenos Aires, (C1121ABG), Argentina; 2 Laboratorio de Retrovirus, Fundación de Ayuda al Inmunodeficiente (FUNDAI), (Uspallata 2272), Buenos Aires, (C1282AEN), Argentina; 3 Virology Section. Francisco Javier Muñiz, Infectious Diseases Hospital, (Uspallata 2272), Buenos Aires, (C1282AEN), Argentina; 4 Servicio de Inmusuprimidos, Ramos Mejia Hospital, (Urquiza 609), Buenos Aires, (C1221ADC), Argentina; 5 St James School of Medicine, (Lake Albert Rd), The Quarter, (A-1 2640), Anguilla

## Abstract

**Background:**

Genetic characterization of HIV-1 in Argentina has shown that BF recombinants predominate among heterosexuals and injecting drug users, while in men who have sex with men the most prevalent form is subtype B.

**Objectives:**

The aim of this work was to investigate the presence of HIV dual infections in HIV-infected individuals with high probability of reinfection

**Study design:**

Blood samples were collected from 23 HIV positive patients with the risk of reinfection from Buenos Aires. A fragment of the HIV gene *pol *was amplified and phylogenetic analyses were performed. Antiretroviral drug resistance patterns of all the sequences were analyzed.

**Results:**

Five dual infections were detected with four patients coinfected with subtype B and BF recombinants and one patient was coinfected with two BF recombinants presenting different recombination patterns. Prolonged infection with a stable clinical condition was observed in the five individuals. Resistance mutation patterns were different between the predominant and the minority strains.

**Conclusions:**

Our results show that HIV dual infection can occur with closely related subtypes, and even with different variants of the same recombinant form in certain populations. Clinical observations showed neither aggressive disease progression nor impact on the resistance patterns in the dually-infected patients.

## Findings

The occurrence of infection with more than one strain of HIV-1 has important implications for understanding HIV transmission and for the development of an AIDS vaccine, as well as the fact that it is leading to many recombinant strains of global epidemiological relevance [1-3].

Resistance concerns also emerged as a key issue for dually infected patients, since they can acquire a resistant strain or generate a multidrug-resistant virus [4]. Nevertheless, the influence of superinfection on resistance evolution is still unclear [5].

The genetic characterization of HIV-1 in Argentina showed that BF recombinant forms are the most prevalent genetic forms among heterosexuals, and among intravenous drug users (IDUs); while subtype B is the most prevalent in men who have sex with men (MSM) [6-8]. Thus, patients with multiple epidemiological risks (e.g. bisexual men and/or IDUs) may be exposed to both subtype B and BF recombinants.

To evaluate the presence of HIV-1 dual infections we selected HIV-1 positive individuals whom presented multiple epidemiological risks for HIV-1 infection, based on confidential interviews regarding sexual behavior, intravenous drug use and medical history, in Buenos Aires, Argentina. Informed consent was obtained from all individuals. Blood samples were collected and serological studies were performed. Total RNA and genomic DNA isolation from plasma and PBMC respectively were performed, (QIAgen, Valencia, CA, USA). A *pol *gene fragment was amplified as described [9], and cloned into the pCR2.1-Topo vector (Invitrogen) or evaluate by single genome amplification (SGA) [9]. The amplicons were then sequenced with Big Dye terminators using an ABI 3100 automated sequencer (Applied Biosystems Inc, Foster City CA). Drug resistance phenotype was determined using the Stanford University HIV Drug Resistance. Sequence alignment was performed using the CLUSTAL × software, followed by a Neighbor-joining method with Kimura's two-parameter model of distance calculation using MEGA 4.1. Recombinant analysis was performed using SimPlot v2.5

**Figure 1:**
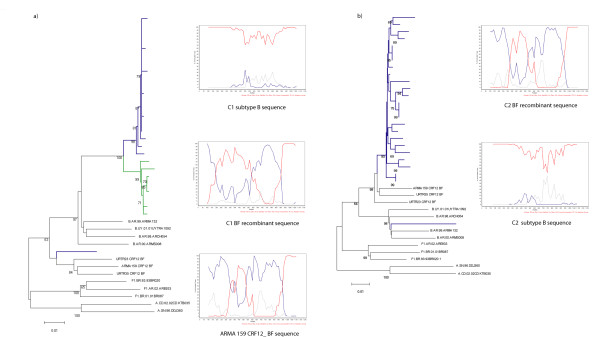
**NJ phylogenetic tree and bootscanning analysis of HIV-1 *pol *region from the sequences of HIV-1 positive individuals with evidence of dual infection**. The trees were constructed with the NJ method and Kimura two-parameter model (ts/tv = 2/1) and they were performed with the complete fragment analyzed. Bootstrap values (1000 resamples) higher than 60 and scale are shown. Bootscanning analysis was performed comparing the sample with the reference sequences: HXB2; WR27, MN and RL42 (subtype B: red line); BR020, VI850, FIN9363 (subtype F: blue line), and U455, SE7253, 92UG037 (subtype A: grey line). A 200 nt window advanced in 20 nt increments was used by the NJ method and Kimura two-parameter model (ts/tv = 2/1). a) Case 1; b) Case 2. Sequences from sample 1 are indicated with blue lines while the sequences from sample 2 are in green.

For case 1 (C1), a dual infection was found in the first sample (C1S1), where 1 out of 21 clones clustered with BF recombinants and the rest with subtype B. The recombinant BF clone exhibited a different *pol *gene recombination pattern than CRF12_BF. However, in C1S2 (2 years later) all of the 12 clones clustered with subtype B references (Figure 1a). The mean of the genetic distances of the B sequences from C1S1 compared to those from C1S2 was 2%, while their distance to other Argentine subtype B sequences was close to 5%, suggesting that C1S2 sequences did not occur due to a new superinfection. This patient was diagnosed in September 1991 and until the sampling period has been asymptomatic. He reported having had heterosexual contacts but for the last 10 years he has only had homosexual intercourse. He had been receiving antiretroviral (ARV) treatment since 1995 (Table 1). The analysis of resistance pattern showed that there were relevant resistance mutations in the RT (Table 2).

**Table 1:** Clinical data from 5 HIV-1 dual infected individuals

Case	Institution	Risk category	Time points	CD4 (cells/μl)	VL (RNA copies/ml plasma)	HAART*
1	R. Mejia Hospital	Bisexual male	S1	NA	2,5 x10^4^	d4T 3TC EFV (failure)
			S2		3,3 x10^5^	ddI ZDV SQV/r (failure)

2	R. Mejia Hospital	Bisexual male	Unique sample	NA	446	No

3	FUNDAI	Bisexual male	Unique sample	> 400	< 50	ZDV 3TC EFV (effective)

4	FUNDAI	Bisexual male- IDU	S1	447	5,7 x10^4^	No
			S2	905	8,2 x10^4^	

5	FUNDAI	IDU	S1	453	NA	ddI D4T NVP**
			S2	497	1,2 x10^4^	

* At the moment of dual infection detection.

** This treatment was two years before sampling.

NA: not available.

**Table 2:** Evolution of antiretroviral resistance mutations in HIV-1 individuals with dual infections

Case	Time points	Primary subtype		Secondary subtype	
		**Prot**	**RT**	**Prot**	**RT**

**C1**		**B subtype**		**BF recombinant (not CRF12)**	
	S1	NONE	M41L/M184V/Y188L/L210W/T215Y^21^ *V106I, H221Y and D67N ^(A)^*	NONE	NONE
	S2	L10I/I15V/M46I/L63P/I84V/I85V/L90M^12^ *M36I and F53L/A71T^(A)^*	M41L/D67N/L210W/T215Y^12^	NON DETECTED	

**C2**		**CRF12_BF**		**B subtype**	
		NONE	NONE	I84V/L90M	M41L/K70E/L210W/T215Y

**C3**		**B subtype**		**CRF12_BF**	
		NONE	K103N^1^	NONE	NONE

**C4**		**CRF12_BF**		**B subtype**	
	S1	I47V^1^	NONE	NONE	NONE
	S2	NONE	K70R^1^	NON DETECTED	

**C5**		**CRF12_BF**		**BF recombinant (not CRF12)**	
	S1	NONE	Y181C/H221Y^2^	NON DETECTED	
	S2	NONE	Y181C^1^	NONE	NONE

A mean of sixteen sequences and/or clones per sample were analyzed with Stanford software. Resistance mutations separeted by bars indicate combination of mutations in the sequences. The number indicated by superscript shows the number of sequences or clones with the corresponding mutation or combination of mutations.

(A) Each mutation in different sequences and carrying the combination of mutations indicated.

In the only sample available from C2, 1 out of 25 sequenced clones clustered with subtype B while the others clustered with BF recombinants (Figure 1b). The BF clones exhibited a similar *pol *gene recombination pattern to CRF12_BF (Figure 1b). This patient was diagnosed in January 2000 and reported having had only homosexual encounters, with some male partners being foreigners and/or HIV-infected. He had no resistance mutations in the BF clones, but subtype B sequence showed major PI resistance mutations (Table 2).

**Figure 2:**
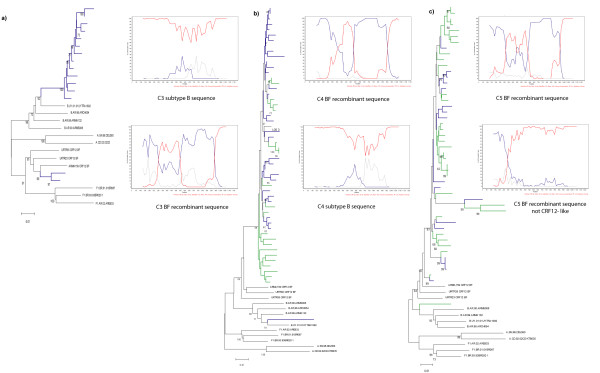
**NJ phylogenetic tree and bootscanning analysis of HIV-1 *pol *region from the sequences of HIV-1 positive individuals with evidence of dual infection**. The trees were constructed with the NJ method and Kimura two-parameter model (ts/tv = 2/1) and they were performed with the complete fragment analyzed. Bootstrap values (1000 resamples) higher than 60 and scale are shown. Bootscanning analysis was performed comparing the sample with the reference sequences: HXB2; WR27, MN and RL42 (subtype B: red line); BR020, VI850, FIN9363 (subtype F: blue line), and U455, SE7253, 92UG037 (subtype A: grey line). A 200 nt window advanced in 20 nt increments was used by the NJ method and Kimura two-parameter model (ts/tv = 2/1). a) Case 3; b) Case 4; c) Case 5. Sequences from sample 1 are indicated with blue lines while the sequences from sample 2 are in green.

In C3, two sequences clustered with the CRF12_ BF, while 13 grouped with B references (Figure 2a). HIV infection was diagnosed in 2000 and the patient declared having had sexual intercourse with members of both sexes. This patient discontinued his treatment without medical indication. But during the sampling period (2004) he was again on HAART and clinically asymptomatic (Table 1).

In C4S1, 20 sequences were obtained from DNA and amplified by SGA and 11 clones from RNA. As shown, one sequence derived from DNA clustered with subtype B references while 19 clustered with CRF12_ BF references (Figure 2b). Recombination as well as similarity to the CRF12_BF was confirmed (Figure 2b). In C4S2, 22 clones clustered with CRF12_BF references. The HIV infection was diagnosed in 1993. Fifteen years prior to the sample collection, he conceded to having had sexual intercourse with men, with only heterosexual relations thereafter. His female partner was an IDU HIV+ and she was also coinfected with HCV and HBV. The patient was clinically asymptomatic during sampling and had never been on HAART (Table 1).

In C5S1, all the sequences clustered with the CRF12_BF with a high bootstrap value (Figure B.e). In C5S2, besides proviral DNA, RNA was also analyzed by cloning. One sequence from S2 amplified by SGA clustered with a BF recombinant showing a different recombination pattern confirmed by bootscanning analysis (Figure 2c). The NJ tree showed that sequences from both samples formed one cluster. HIV infection in this patient was diagnosed in 1998. He reported being an IDU as his only risk factor and was coinfected with HCV. He had always had high CD4 T-cell counts and was clinically asymptomatic at the time of the study (Table 1). He had received HAART therapy but he interrupted it two years before the first sample was taken (Table 2).

It has been observed that several drug resistance-associated mutations in the *pol *region of BF recombinant variants include mutations which are in fact natural polymorphisms of the BF strains [10,11]. Therefore, we only considered major PI-resistance associated mutations to categorize viral variants as resistant. Among the B sequences in C1, there were multiple resistance mutations to NRTI and NNRTI, but the unique BF sequence was susceptible. Hence, the selective pressure of the treatment could be responsible for the predominance of subtype B [12]. Moreover, the changes in the resistance patterns from C1S1 to C1S2 completely mirrored the treatment change (Tables 1 and 2). The fitness costs on the persistence of M184V in the absence of selective pressure has been extensively studied [13] and it has been observed that viruses carrying the TAMs M41L, L210W and T215Y have higher fitness than those with M184V [14]. Therefore, between C1S1 and C1S2, M184V did not persist in the viral population, while the TAMs did. A similar rationale can support the loss of NNRTI resistance-associated mutations. Finally, since the presence of at least three TAMs is associated with cross-resistance to ddI [14], the new HAART scheme actually worked as monotherapy leading to the selection of multiple PI resistance-associated mutations.

In C2, although he had never received ARV therapy, subtype B strain clone was drug-resistant. This virus could have been acquired from a person with a resistant strain, and thus become the minority one because of its lower fitness [15]. Also, in C4 only one sequence from C4S1 carried a mutation to PR while one sequence from C4S2 had one mutation to RT. Since C4 had never received ART, these minority viral quasispecies might have disappeared. The evolution of his HIV disease had not been aggressive over time, but the RNA and proviral DNA sequences from C4S1 intermingled among the proviral sequences from S2, indicating active replication [16].

HIV-1 superinfections have been described after treatment interruption, as in C3, the minority CRF12_BF virus did not show any resistance mutations, it could be hypothesized that it might have been acquired after treatment interruption [17] (Table 2).

C5 had been under HAART, but treatment had been suspended due to medical advice. As with C4, active replication was observed [16]. The putative superinfection was likely coincidental with the relapse of intravenous drug use; which may explain the fact that the secondary virus was a BF recombinant, since these are common among IDUs [7]. Previous HAART probably explains the resistance mutations found in sequences of the predominant virus. Since the secondary viral sequence did not show any resistance mutations, as in C3, treatment interruption might have been a major predisposing factor leading to superinfection [17]. This individual could have acquired the second infection at least 8 years after the first one. In this regard, recent studies have found lack of protection against superinfection in chronic HIV-infected individuals as well as no significant deficits in neutralizing antibodies response [18]. In C5, the evaluation of the recombination patterns of each sequence and the analysis of partial trees performed taking into account the breakpoint of the superinfecting strain, suggest that, to the best of our knowledge, this is the first case of HIV dual-infection with different BF recombinants (Figure 2c and data not shown).

The link between dual infection and recombinant forms has been already shown [19,20], and since a high viral load level is important in transmission, it could be that recombinants are generated in individuals who also develop higher viral loads and readily transmit them. Eventually, superinfection and double infection would be expected to increase the complexity of viral genotypes circulating in the population [21,22].

In this report, we described five cases of dual infections from 23 individuals with multiple epidemiological risks. This study underlines the frequency of dual infection in high risk populations, but additional research will be needed to interpret superinfection in the context of anti-HIV-1 immunity and vaccine development.

## Competing interests

The authors declare that they have no competing interests.

## Authors' contributions

GA, CE and AC were equally responsible for the design, cloning, viral characterization, and writing of the manuscript. JA, AP, and JKC were responsible for viral characterization and resistance studies. DP, MBB, SG, MW, ML and JB were responsible for the clinical data from patients. LMP was responsible for the design and writing of the manuscript. All authors read and approved the final manuscript.
